# Effect of Dead Sea Climatotherapy on Psoriasis; A Prospective Cohort Study

**DOI:** 10.3389/fmed.2020.00083

**Published:** 2020-03-18

**Authors:** Thomas Emmanuel, Dorte Lybæk, Claus Johansen, Lars Iversen

**Affiliations:** Department of Dermatology, Aarhus University Hospital, Aarhus, Denmark

**Keywords:** psoriasis, Dead Sea, climatotherapy, heliotherapy, cohort, Ein Gedi

## Abstract

**Background:** Dead Sea climatotherapy (DSC) is a treatment option for psoriasis in Denmark. However, the response to DSC has not been particularly well studied.

**Aim:** We sought to determine effectiveness and response duration of DSC on psoriasis-related outcome parameters.

**Methods:** Eighteen patients participated in a 4-week treatment program in Ein Gedi in Israel. Treatment, consisting of sun exposure and bathing, was individualized.

**Results:** DSC was associated with a mean 13.0-point reduction (88%) in Psoriasis Area and Severity Index and a mean reduction of 2.3 (76.7%) on the 5-point Investigator's Global Assessment Scale. Furthermore, patients' quality of life improved measured by the Dermatology Quality of Life Index and EuroQol 5D index values. The mean time from treatment end to reappearance of visible skin symptoms was 93.8 days (SD: 62.5, range: 31–219 days).

**Conclusions:** Our results confirm that DSC has an immediate effect on skin manifestations and improves quality of life, but long-term disease control is not observed.

## Introduction

Psoriasis is a chronic skin disease affecting ~1–3% of the western population (1). It has a wide range of clinical phenotypic appearances, ranging from pruritic scaly red plaques on the skin to isolated affection of the nails (2). Treatment modalities range from topical treatment and photo therapy to systemic treatment with small molecules and biological therapies (3). Treatment of skin diseases using UV light has been used since 1903 when Niels Ryberg Finsen won the Nobel prize for reporting on the effective treatment of tuberculosis of the skin (4). Phototherapy, i.e., treatment exclusively using light, is usually supplemented with balneotherapy i.e., baths in salt water, collectively termed balneophototherapy (BPT). In addition, a change of humidity and air, and a change in barometric pressure is added and then collectively termed climate therapy or climatotherapy (5). Climatotherapy is a safe treatment option, and beneficial effects on psoriasis have been reported (6, 7). Climatotherapy has also been shown to improve quality of life (QoL) (8). In some countries, it is an alternative to medication for psoriasis with treatment centers located all over the world (9). The proportion of psoriasis patients attending a university hospital dermatology clinic and having used climatotherapy at some time during the course of their treatment varies considerably across the world; from 8% in the Nordic countries to 46% in the Middle East (10). Danish psoriasis patients have mainly received climatotherapy at Ein Gedi at the Dead Sea in Israel located at 31°N, 35°E. With a location ~400 meters below sea level, patients are exposed to salt and minerals and prolonged sun exposure while exposure to cancer-inducing ultraviolet B (UVB) radiation from the sun is minimal (11–13). This combination is unique among treatment centers. Dead Sea climatotherapy (DSC) has a long-standing history, and Denmark was one of the first countries to test it in psoriasis patients (14). DSC is particularly relevant where other therapies are contraindicated or have failed. Patients treated in Israel constitute a subgroup of psoriasis patients that has not previously been much studied.

In the present study, we therefore investigated the effect of DSC on psoriasis by measuring psoriasis-related outcomes before, immediately after, and at first visible signs of psoriasis after completion of DSC.

## Materials and Methods

### Study Design and Population

Eighteen Danish patients were enrolled in this prospective cohort study. The study size was based on feasibility. The inclusion criteria were referral for DSC. Referral was a shared decision between the treating dermatologist and the patient. The date of enrolment of the first- and last patient was on the 18th of February 2015 and 2nd of September 2015, respectively. All patients referred to climatotherapy for the given enrolment period were given the possibility of being included in this study. The last follow-up visit of the last patient was on the 5th of January 2016. Patients were allowed to use their usual treatments at baseline. No formal exclusion criteria were applied though contraindications for selection for DSC include photo-aggravated systemic diseases or dermatoses, skin malignancies, acute skin infections, non-controlled concomitant diseases, and lack of compliance with instructions regarding sun exposure and bathing.

All patients were sent on a 4-week treatment program at Ein Gedi at the Dead Sea in Israel. Here, they received individualized treatment consisting of 28 days of bathing and escalating doses of UVB exposure according to skin type and UV index. Treatment was supplemented with educational lectures and discussions about psoriasis and QoL, comorbidities, manifestations, and various treatments. A healthy lifestyle consisting of physical activity and a healthy diet was stressed. Group discussions were instituted for sharing ways to manage psoriasis. Individual and group-based education, guidance, and daily training were provided by a nurse and a physiotherapist. Treatment-related costs were fully covered by the Danish Medical Insurance System. The study was an open-label, single-arm cohort study with up to three repeated measures for a maximum of 8 months. Patients were to be assessed by the same dermatologist 1 week before (baseline) and immediately after (visit 1) climatotherapy, and at the first visible sign of psoriasis reappearance (visit X). At these assessments, we obtained data on Psoriasis Area And Severity Index (PASI), 5-point Investigator's Global Assessment (IGA) Scale, Nail Psoriasis Severity Index (NAPSI), Body Mass Index (BMI), waist circumference, blood pressure, and pulse; and patients answered the following questionnaires: Nail Assessment in Psoriasis and Psoriatic Arthritis (NAPPA), Dermatology Quality of Life Index (DLQI), and EuroQol−5 Dimensions−3 Levels (EQ-5D-3L) [consisting of the EQ-5D descriptive system and EQ-VAS (EuroQol—Visual Analog Scale)]. Patients were encouraged to contact the study coordinators as soon as their psoriasis skin symptoms reappeared. They would then be booked for an appointment with the dermatologist.

### Statistics

Figure and statistical analysis was performed in SigmaPlot v. 14.0 (Systat Software, Chicago, IL, USA). Normality was tested using the Shapiro-Wilk normality test. For parametric data a one-way repeated measures analysis of variance with Bonferroni *post hoc* test was used to compare results from different visits. For non-parametric data a Friedman repeated measures analysis of variance on ranks was used. Missing data were not included in the statistical analysis. All *P*-value calculations were two-sided, and a *P*-value of <0.05 was considered significant.

## Results

**Figure 1:**
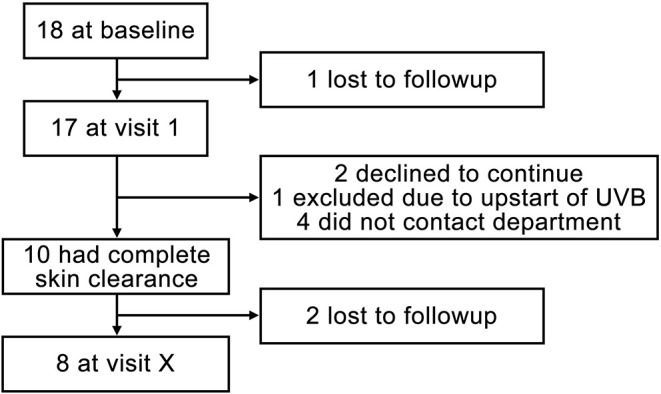
Flowchart of included patients.

**Figure 2:**
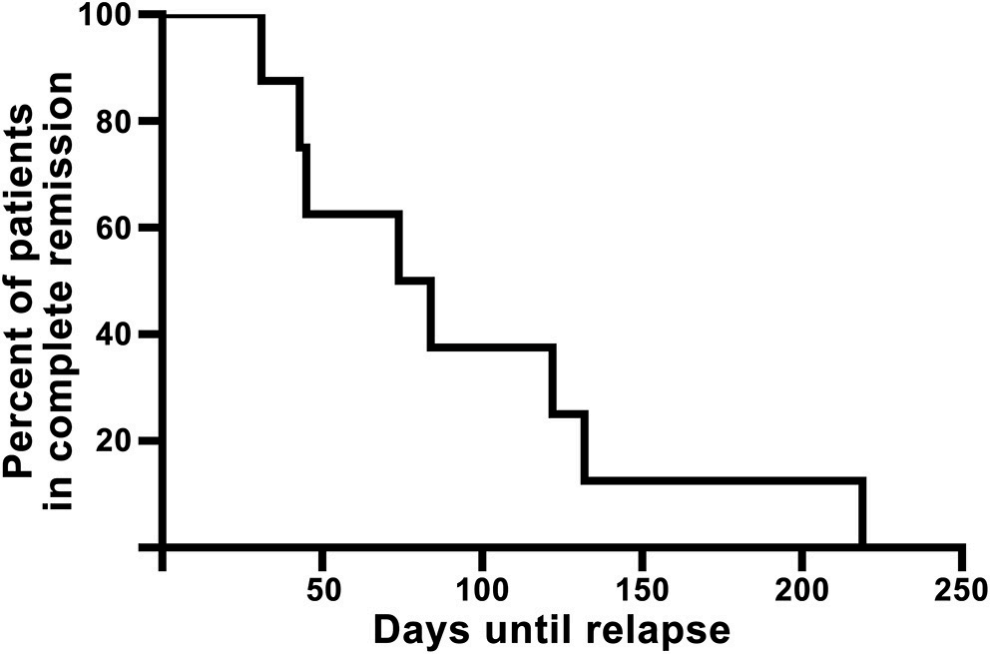
Kaplan-Meier curve of the eight full responders showing time from end of treatment until reappearance of visible psoriasis (visit X).

All 18 patients completed the planned treatment. Figure 1 shows a flowchart of included patients. In summary, 17 patients were seen at visit 1; of 10 (55.6%) patients achieving complete skin clearance after treatment, eight were seen at visit X. Demographic characteristics at baseline can be seen in Table 1. Full individual demographic characteristics can be seen in Table S1. No side effects of treatment were noted. No significant differences in BMI, waist circumference, blood pressure, or pulse were observed between the visits (data not shown). The time from baseline to visit 1 was 46.5 days (SD: 7.8, range: 32–57). The time from baseline to departure was mean 13.6 days (SD: 5.7, range: 2–25). The mean time from end of treatment to visit 1 was 4.4 days (SD: 4.4, range: 0–17). The mean time from visit 1 to visit X was 90.9 days (SD: 63.7, range: 21–216). The mean time from end of treatment to reappearance of skin symptoms was 93.8 days (SD: 62.5, range: 31–219 days). A Kaplan-Meier curve showing time to reappearance of skin symptoms among full responders can be seen on Figure 2.

**Table 1:** Demographic and clinical characteristics of the cohort at baseline. (*n* = 18).

	**Baseline**	
Sex, *n* (%)	–	
Male	12 (67)	
Female	6 (33)	
Age, years, mean (SD) [range]	52.2 (12.2)	[25-71]
Body Mass Index (kg/m2), mean (SD) [range]	28.5 (6.8)	[19.5–46.1]
Waist circumference (cm), mean (SD) [range]	104.7 (16.3)	[81.5–142]
Duration of psoriasis, years, mean (SD) [range]	34.2 (17.8)	[8-60]
Treatment duration, days, mean (SD) [range]	28.8 (3.2)	[24-38]
Previous climate therapy, *n* (%)	–	
Yes	15 (83)	
Number of climate treatments, mean (SD) [range]	11.2 (7.0)	[1-25]
Type of disease, *n* (%)	–	
Nail psoriasis	18 (100)	
Psoriatic arthritis	7 (39)	
Psoriasis-related comorbidities, *n* (%)	–	
Anxiety	2 (11)	
Asthma	1 (6)	
Chronic obstructive pulmonary disease	1 (6)	
Depression	1 (6)	
Diabetes	5 (28)	
Hypercholesterolemia	2 (11)	
Hypertension	6 (33)	
Osteoarthritis	2 (11)	
Medication at baseline, *n* (%)	8 (44)	
Topical steroids	8 (44)	
UVB	2 (11)	
No medication	10 (56)	
Treatment for nail psoriasis 12 months prior to or during baseline, *n* (%)	4 (22)	
Family history of psoriasis, *n* (%)	14 (78)	
No family history	3 (17)	
Smoking, *n* (%)	2 (11)	
Alcohol, *n* (%)	0 (0)	
Sick leave due to psoriasis, *n* (%)	0 (0)	
Professional educational requirements, *n* (%)	–	
High school	11 (61)	
Post-secondary diploma	4 (22)	
University degree	0 (0)	
Prior treatments	–	
Topical Steroids, *n* (%)	17 (94)	
Light therapy, *n* (%)	15 (83)	
Tar, *n* (%)	7 (39)	
Oral systemic, *n* (%)	13 (72)	
Biologic, *n* (%)	4 (22)	

Results from clinical and patient questionnaire scores can be seen on Table 2. Full individual data can be seen in Tables S2, S3. From baseline to visit 1, the results were for the most part similar for each clinical and questionnaire measure; mean values improved for all measures, but improvements were significant only for PASI, IGA, DLQI, and EQ-5D index values. All patients had a PASI reduction (mean 13.0, equivalent to 88% reduction). The mean IGA reduction was 2.3 (76.7%), DLQI showed a mean improvement of 11.9 (85.6%) and EQ-5D index values were improved by 0.11 (13.9%).

**Table 2:** Results from clinical and patient questionnaire scores.

**Clinical evaluation**	**Baseline**	**Visit 1**	**Visit X**	***P*-values**
PASI, mean (SD), [range], {values}	14.8 (5.4), [7.3–27], {15}	1.8 (3.1), [0–9.7], {15}	5.8 (3.1), [2.7–9.9], {6}	**a** **≤** **0.001** b = 0.120 **c** **=** **0.002**
IGA, mean (SD), [range], {values}	3 (0.4), [0–4], {17}	0.7 (1.0), [0–3], {15}	1.9 (0.9), [1-3], {7}	**a** **≤** **0.001** b = 0.050 **c** **≤** **0.001**
NAPSI Hands + Feet, mean (SD), [range], {values}	58.1 (54.0), [0–160], {15}	54.6 (47.6), [0–124], {15}	23.2 (38.8), [0–92], {5}	NS
NAPSI Hands, mean (SD), [range], {values}	28.8 (24.4), [0–80], {14}	27.1 (22.4), [0–62], {14}	15 (23.4), [0–56], {5}	NS
NAPSI Feet, median (interquartile range), {values}	240 (3–73), {13}	16 (2–60), {15}	2 (0–19.5), {5}	NS
**Self-reported questionnaires**				
NAPPA-Global, mean (SD), [range], {values}	1.0 (0.9), [0–3], {18}	0.6 (0.8), [0–2.3], {16}	1.1 (1.0), [0–2.7], {8}	NS
NAPPA-Signs, mean (SD), [range], {values}	1.6 (1.2), [0–3.7], {18}	0.9 (1.1), [0–3.3], {16}	0.9 (1.0), [0–2.4], {7}	NS
NAPPA-Stigma, mean (SD), [range], {values}	0.8 (3.0), [0–2.4], {18}	0.6 (0.8), [0–2.4], {16}	0.7 (1.1), [0–2.9], {7}	NS
NAPPA-Everyday Life, mean (SD), [range], {values}	0.8 (0.9), [0–2.9], {18}	0.4 (0.6), [0–2.1], {16}	0.9 (1.0), [0–2.4], {7}	NS
DLQI, mean (SD), [range], {values}	13.9 (7.6), [2-28], {18}	2.0 (3.5), [0–13], {16}	7.4 (6.2), [0–16], {7}	**a** **≤** **0.001** **b** **=** **0.024** c = 0.114
EQ-5D index values, mean (SD), [range], {values}	0.79 (0.09), [0.63–1.00], {18}	0.90 (0.13), [0.64–1.00], {16}	0.82 (0.10), [0.70–1.00], {6}	**a** **=** **0.008** b = 0.134 c = 1.000
EQ-VAS, mean (SD), [range], {values}	61.0 (16.1), [25-95], {17}	76.0 (23.1), [20-100], {16}	59.6 (14.3), [40–75], {7}	NS

*a (baseline compared to visit 1), b (visit 1 compared to visit X), c (baseline compared to visit X). Bold signifies statistical significance. NS, No significant difference among treatment groups; PASI, Psoriasis Area Severity Index; IGA, 5-point Investigator's Global Assessment; NAPSI, Nail Psoriasis Severity Index; NAPPA, Nail Assessment in Psoriasis and Psoriatic Arthritis; DLQI, Dermatology Quality of Life Index; EQ-5D-3L, EuroQol−5 Dimensions−3 Levels; EQ-VAS, EuroQol—Visual Analog Scale*.

Neither NAPSI, NAPPA nor EQ-VAS was significantly improved between baseline and visit 1.

## Discussion

DSC has been well established as a treatment option affecting not only physical appearance but also patients' QoL (8, 15, 16). These effects are, unfortunately, not everlasting. Remission times vary in the literature due to various definitions of remission and relapse (17). In our study, remission time was defined as the skin symptom-free interval. Ten patients achieved complete psoriasis clearance and eight of those had confirmed re-emergence of psoriasis within the study period. Among those patients, the mean symptom-free interval was 93.8 days (13.4 weeks). One study using a German cohort reported remission lasting 24.9 weeks (16). Another study showed complete remission in 74% of the patients after 6 months and a mean remission of 196 days (15). Additionally, one study showed remission lasting 100 days but offered no clear definition of remission (18). In our study, the high mean PASI and IGA scores at visit X of 5.8 (SD: 3.1, range: 2.7–9.9) and 1.9 (SD: 0.9, range: 1–3), respectively, could suggests a delay between first experiencing cutaneous symptoms and then contacting the department. This could lead to an overestimation of the duration of remission. However, the results presented here still suggest that the skin symptom-free interval is comparable to what is reported by others.

We observed a PASI improvement of 88% and complete clearance in 55.6% of patients at visit 1. This is in accordance with previous studies on DSC, which report improvement ranging from 81.5 to 95.5% and complete clearance ranging from 48 to 70% immediately after DSC (7, 16, 18–20). The IGA reduction further substantiates the short-term effectiveness of DSC.

BPT is an effective treatment option for psoriasis and an alternative to DSC. BPT is performed at centers located worldwide, is known to improve QoL and has shown efficacy in randomized clinical trials (21, 22). Dawe et al. (23) conducted a randomized trial on 60 patients on the effect of narrowband-UVB alone and in combination with localized Dead Sea salt soaks. Among patients who achieved complete remission no difference in remission times was found between treatments. However, the psoriasis severity score fell significantly more from baseline to end of treatment sessions when comparing combination to narrowband-UVB alone (23). The study by Klein et al. (24) included 356 patients and investigated the effect of UVB alone and in combination with a whole-body Dead Sea salt bath. A relative PASI reduction of 67.4% was observed in the combination group after the 35th treatment. Clearance, defined as PASI improvement of at least 75% from baseline, was not observed in any of the participants (24). Treatment time for the studies by Dawe et al. (23) and Klein et al. (24) was ~12 and 11 weeks, respectively. A randomized study by Léauté-Labrèze et al. (25) conducted on 71 psoriasis patient compared spa water alone; phototherapy alone and combination. They observed a PASI reduction of 64 and 55% in the phototherapy and combination group at 3 weeks, respectively. However, no significant difference between the two groups was observed (25). Two larger German randomized control trials including 143 and 1,241 patients also investigated the effect of BPT on psoriasis (26, 27). Brockow et al. (26) studied the effect of concentrated saline spa water baths followed by UVB and found a median PASI reduction of 66% after 6 weeks of treatment. Forty-nine percentage had PASI < 5 after intervention and 31% had PASI < 5 after 3 months (26). Schiener et al. found a median PASI improvement of 76% after a maximum of 8 weeks of treatment (27). A retrospective clinical study on 174 patients investigating the effect of BPT on PASI and QoL found an improvement in PASI of 70.9% and QoL of 51.2% after 30 treatments (~6 weeks) (28). We report a faster treatment response than studies conducted on BPT. BPT, however, is often performed in clinics closer to the patient's home and therefore does not require relocation to more disease favorable locations.

New biological treatments are now being used for treating moderate-to-severe plaque psoriasis. Biologics are a heterogenous group of agents with different molecular targets and efficacy, however in long-term clinical trials and epidemiologic studies, their safety profile and efficacy are acceptable (29–31). Moreover, short-term data on biologics are robust, with up to 70.7% of patients achieving more than 90% reduction in PASI and up to 89.7% achieving a PASI 75 response after 12 weeks of treatment dependent on the specific biologic and dose used (31). Regarding the longer-term effects of biologics, a Swedish study found that switching to biologics was associated with a sustained effect at 10 years with a PASI reduction of 82.8%, a DLQI improvement of 85.7%, and an EQ-5D improvement of 11.0% (32). A study conducted on patients with moderate-to-severe psoriasis comparing the effect of biologics with that of systemic, topical, or light treatment found that biologics resulted in an average mean reduction in DLQI of 6.6 at week 24 (33). A double blinded randomized study comparing ixekizumab with guselkumab found that 35 out of 520 (7%) of ixekizumab- and 7 out of 507 (1%) of guselkumab treated patients achieved a PASI 100 response after 4 weeks and a median PASI percentage improvement of at least 75% for ixekizumab and 50% for guselkumab (34). We report short term results after 4-weeks of treatment that are faster or comparable with the newest biologics. However, a strength of continuous usage of newer biologics are that their effect lasts longer; moreover, they have a positive effect on psoriasis-related parameters and, unlike DSC, do not require patients to complete a 4-week extensive treatment program.

DSC is known to improve QoL parameters (8, 16). We observed an improvement in DLQI and EQ-5D index values after treatment, further substantiating this observation. Nail psoriasis is a subset of psoriasis that usually responds poorly to conventional therapy. To our knowledge, the present study is the first study to include nail assessments as a separate measure. We found no significant improvement in NAPSI or NAPPA parameters after treatment; but due to the slow growth rate of nails, we would not expect nail parameters to improve until several months after treatment. Interestingly, we observed a non-significant improvement in NAPSI between baseline and visit X, substantiating this expectation of a trend in objective nail improvement.

We report no adverse effects, though it has been shown that the risk of non-melanoma skin cancer is almost increased 5-fold for Danish psoriasis patients compared with the background population (35). Another study using an Israeli cohort found that treatment-related solar damage might give rise to chronic solar damage such as wrinkles and solar lentigines, though no increased risk of non-melanoma skin cancers was found (36). This suggest that patients' ethnicity and treatment side effects should be taken into consideration when referring patients for DSC.

A strength of this study is the plethora of parameters used to appraise the effect of DSC, giving us a full and comprehensive view of psoriasis morbidity. Additionally, the same dermatologist evaluated all patients throughout the study, thereby eliminating inter-evaluator bias. The limitations of this study include the small number of patients, the small number of data points, and the lack of a control group. However, blinding and randomization are difficult to institute in this kind of intervention. The open-label design used here might therefore contribute to an overestimation of the results. In this study, we cannot conclude whether the positive effect on psoriasis morbidity is sustained beyond visit X. Therefore, future studies should include larger sample sizes, more follow-up points, and longer follow-up times to further elucidate both the short-term and long-term effects of DSC on psoriasis.

In conclusion, this study confirms that DSC can be beneficial in the short term for treatment of both psychological and dermatological aspects of psoriasis, but the effects are not long lasting.

## Data Availability Statement

Datasets generated for this study are included either in the article/Supplementary Material or are available on request.

## Ethics Statement

All patients signed an individual written informed consent form. The Central Denmark Region Committees on Health Research Ethics committee waived the requirement for ethical approval for this study on human participants due to the study being a quality control study, in accordance with the national legislation and the institutional requirements. Procedures were conducted in accordance with the Helsinki Declaration of 1975 as revised in 2013.

## Author Contributions

TE, DL, CJ, and LI participated in the design of the study, monitoring the study, and wrote sections of the manuscript. TE conducted the statistical analysis and wrote the first draft of the manuscript. All authors contributed to manuscript revision, read, and approved the submitted version.

### Conflict of Interest

The authors declare that the research was conducted in the absence of any commercial or financial relationships that could be construed as a potential conflict of interest.
