# Supplementary material for: Effect of Dead Sea Climatotherapy on Psoriasis; A Prospective Cohort Study
Source: Front Med (Lausanne). 2020 Mar 18;7:83. doi: 10.3389/fmed.2020.00083 (PMC7093374; doi:10.3389/fmed.2020.00083)
Supplement: Supplementary file 1 [file Table_1.DOCX]

| Patient no. | 1 | 2 | 3 | 4 | 5 | 6 | 7 | 8 | 9 | 10 | 11 | 12 | 13 | 14 | 15 | 16 | 17 | 18 |
| --- | --- | --- | --- | --- | --- | --- | --- | --- | --- | --- | --- | --- | --- | --- | --- | --- | --- | --- |
| Age, *yr* | 57 | 64 | 46 | 38 | 59 | 25 | 52 | 45 | 71 | 57 | 51 | 57 | 50 | 57 | 42 | 35 | 71 | 62 |
| Gender | F | M | M | M | F | M | M | M | F | M | F | F | M | M | M | M | M | M |
| BMI, *kg/m2* | 34.2 | - | 20.8 | - | 25.9 | 25.8 | 29.6 | 25.7 | 23.9 | 33.4 | 19.5 | 34.1 | 46.1 | 21.4 | 33.2 | 23.8 | 31.2 | 27.7 |
| Waist circumference, *cm* | 123 | - | 91 | 104 | 96 | 98 | 108 | - | 94 | 131 | 81.5 | 116 | 142 | 89 | 106.5 | 91 | 103 | 103 |
| Duration of psoriasis, *yr* | 50 | 50.5 | 29 | 23 | 41 | 10 | 28 | 17 | 56 | 12 | 48 | 45 | 39 | 8 | 34 | 9 | 60 | 56 |
| Previous climate therapy, *n* | 11 | 17 | 7 | 8 | - | 1 | 16 | 10 | 0 | 5 | 20 | 11 | 12 | 6 | 18 | 1 | 25 | - |
| Nail psoriasis, *n* | Yes | Yes | Yes | Yes | Yes | Yes | Yes | Yes | Yes | Yes | Yes | Yes | Yes | Yes | Yes | Yes | Yes | Yes |
| Psoriasis arthritis, *n* | Yes | No | No | Yes | No | No | Yes | No | No | No | Yes | Yes | No | Yes | No | No | No | Yes |
| Comorbidities | Anxiety, depression, DM, hypertension, osteoarthritis | COPD | None | Hypercholesterolemia, hypertension, osteoarthritis | None | None | None | Anxiety, DM, hypercholesterolemia, hypertension | Asthma, hypertension | None | None | DM, Hypertension | DM, hypertension | None | DM | None | None | None |
| Medication at baseline | None | Topical steroids | None | Topical steroids | Topical steroids, UVB | Topical steroids | Topical steroids | None | None | None | Topical steroids | Topical steroids | None | None | Topical steroids, UVB | None | None | None |
| Treatment for nail psoriasis 12 months prior to or during baseline | No | Yes | No | Yes | No | Yes | No | No | No | No | - | No | No | No | Yes | - | No | No |
| Family history of psoriasis | - | Yes | Yes | Yes | Yes | Yes | Yes | Yes | Yes | No | Yes | Yes | Yes | No | Yes | No | Yes | Yes |
| Smoker | - | - | - | Yes | Yes | - | - | No | Ex | - | - | - | - | - | - | - | - | - |
| Alcohol | - | - | - | - | - | - | - | - | No | - | No | - | - | No | - | - | - | - |
| Sick leave due to psoriasis | No | - | - | No | No | No | - | - | No | - | - | - | - | No | No | - | No | No |
| Prior treatments | Topical steroids,  UVB,  tar, MTX, Enbrel, Humira, Remicade, climatotherapy | Topical steroids, UVB, neotigason, MTX, climathotherapy | Topical steroids, UVA, UVB, PUVA, neotigason, MTX, tar, climathotherapy | Topical steroids, UVB, MTX, climathotherapy | - | Topical steroids, UVB, bucky, climathotherapy | Topical steroids, MTX, climathotherapy | Topical steroids, UVB, MTX, climathotherapy | Topical steroids, tar, bucky, MTX | Topical steroids, UVB, uncategorized systemic, climatotherapy | Topical steroids, tar, MTX, sandimmun, uncategorized biological, climathotherapy | Topical steroids, UVB, climathotherapy | Topical steroids, UVB, PUVA, tar, MTX, climatotherapy | Topical steroids, bucky, climathotherapy | Topical steroids, UVB, tar, MTX, neotigason, Humira, Enbrel, Remicade, climathotherapy | Topical steroids, UVB, bucky, climathotherapy | Topical steroids, UVB, MTX, climathotherapy | Topical steroids,  carbon-arc light, tar, MTX, Humira, Cimzia, |
| Days in remission | 84 | - | 122 | 31 | - | - | 219 | 74 | - | - | - | 132 | - | - | 43 | - | - | 45 |

Table S1. Individual demographic data of the patient cohort at baseline. COPD=chronic obstructive pulmonary disease, DM=Diabetes mellitus, MTX=methotrexate.
