# Supplementary material for: Effect of Dead Sea Climatotherapy on Psoriasis; A Prospective Cohort Study
Source: Front Med (Lausanne). 2020 Mar 18;7:83. doi: 10.3389/fmed.2020.00083 (PMC7093374; doi:10.3389/fmed.2020.00083)
Supplement: Supplementary file 2 [file Table_2.DOCX]

| Patient no. | 1 | 2 | 3 | 4 | 5 | 6 | 7 | 8 | 9 | 10 | 11 | 12 | 13 | 14 | 15 | 16 | 17 | 18 |
| --- | --- | --- | --- | --- | --- | --- | --- | --- | --- | --- | --- | --- | --- | --- | --- | --- | --- | --- |
| PASI baseline | 11.5 | - | - | 10.6 | 14.3 | 8.9 | 11.6 | 23.6 | 13.2 | 16.5 | 7.3 | 17 | 18.7 | - | 27 | 17.6 | 13 | 11.4 |
| PASI visit 1 | 0 | 3.7 | 0 | 0 | 1.8 | - | 0 | 0 | 0 | 9.7 | 0 | 0 | 0 | - | 4.8 | 6.6 | - | 0 |
| PASI visit X | 9.3 | - | 4.5 | 2.7 | - | - | - | 3 | - | - | - | - | - | 9.9 | - | - | - | 5.6 |
| IGA baseline | 3 | 0 | 2 | 3 | 0 | 2 | 3 | 0 | 2 | 3 | 0 | 2 | 3 | 0 | 2 | 3 | 0 | 2 |
| IGA visit 1 | 3 | 2 | - | 3 | 2 | - | 3 | 2 | - | 3 | 2 | - | 3 | 2 | - | 3 | 2 | - |
| IGA visit X | 3 | 0 | 1 | 3 | 0 | 1 | 3 | 0 | 1 | 3 | 0 | 1 | 3 | 0 | 1 | 3 | 0 | 1 |
| NAPSI Hands + Feet baseline | 2 | 120 | 13 | 63 | 7 | 160 | 110 | 22 | 113 | 50 | - | 6 | 24 | - | - | 0 | 56 | 125 |
| NAPSI Hands + Feet visit 1 | 14 | 120 | 14 | 106 | 0 | 124 | 52 | 35 | 16 | - | 88 | 4 | 49 | - | 73 | 0 | - | 124 |
| NAPSI Hands + Feet visit X | 6 | - | 14 | 92 | 0 | - | - | 4 | - | - | - | - | - | - | - | - | - | - |
| NAPSI Hands baseline | - | 40 | 11 | 53 | 7 | 80 | 50 | 22 | 47 | 10 | - | 2 | 4 | - | - | 0 | 32 | 45 |
| NAPSI Hands visit 1 | 12 | 40 | 12 | 54 | 0 | 62 | 40 | 35 | - | - | 28 | 2 | 6 | - | 37 | 0 | - | 60 |
| NAPSI Hands visit X | 3 | - | 12 | 56 | 0 | - | - | 4 | - | - | - | - | - | - | - | - | - | - |
| NAPSI Feet baseline | 2 | 80 | 2 | 10 | - | 80 | 60 | - | 66 | 40 | - | 4 | 20 | - | - | 0 | 24 | 80 |
| NAPSI Feet visit 1 | 2 | 80 | 2 | 52 | 0 | 62 | 12 | 0 | 16 | - | 60 | 2 | 43 | - | 36 | 0 | - | 64 |
| NAPSI Feet visit X | 3 | - | 2 | 36 | 0 | - | - | 0 | - | - | - | - | - | - | - | - | - | - |

Table S2. Individual clinical scores. PASI = Psoriasis Area Severity Index. IGA = 5-point Investigator's Global Assessment. NAPSI = Nail Psoriasis Severity Index.
