# Supplementary material for: Effect of Dead Sea Climatotherapy on Psoriasis; A Prospective Cohort Study
Source: Front Med (Lausanne). 2020 Mar 18;7:83. doi: 10.3389/fmed.2020.00083 (PMC7093374; doi:10.3389/fmed.2020.00083)
Supplement: Supplementary file 3 [file Table_3.DOCX]

Table S3. Individual questionnaire scores. NAPPA = Nail Assessment in Psoriasis and Psoriatic Arthritis. DLQI = Dermatology Quality of Life Index. EQ-5D = EuroQol - 5 Dimensions. EQ-VAS = EuroQol - Visual Analogue Scale)).

| Patient no. | 1 | 2 | 3 | 4 | 5 | 6 | 7 | 8 | 9 | 10 | 11 | 12 | 13 | 14 | 15 | 16 | 17 | 18 |
| --- | --- | --- | --- | --- | --- | --- | --- | --- | --- | --- | --- | --- | --- | --- | --- | --- | --- | --- |
| NAPPA-Global baseline | 0.2 | 1.7 | 0.0 | 2.9 | 0.1 | 1.7 | 0.6 | 3.0 | 1.1 | 0.9 | 1.9 | 0.3 | 1.0 | 0.6 | 0.4 | 0.8 | 1.0 | 0.9 |
| NAPPA-Global visit 1 | 0.9 | 0.0 | 1.9 | 0.0 | 1.8 | 0.0 | 2.3 | 0.4 | 0.3 | 1.0 | 0.0 | 0.0 | 0.5 | 0.1 | 0.2 | - | - | 0.6 |
| NAPPA-Global visit X | 0.8 | - | 0.0 | 2.1 | 1.8 | - | 0.5 | 2.7 | - | - | - | - | - | 0.1 | - | - | - | 1.0 |
| NAPPA-Signs baseline | 0.7 | 3.5 | 0.0 | 3.7 | 0.2 | 3.3 | 0.7 | 3.0 | 2.3 | 1.3 | 1.0 | 0.5 | 2.3 | 0.7 | 0.2 | 1.8 | 2.0 | 1.7 |
| NAPPA-Signs visit 1 | 1.8 | 0.0 | 2.8 | 0.0 | 3.3 | 0.0 | 2.3 | 0.8 | 1.0 | 0.7 | 0.0 | 0.0 | 0.2 | 0.0 | 0.3 | - | - | 1.3 |
| NAPPA-Signs visit X | 0.9 | - | 0.0 | 2.1 | - | - | 0.4 | 2.4 | - | - | - | 0.0 | - | - | - | - | - | 0.4 |
| NAPPA-Stigma baseline | 0.0 | 1.0 | 0.0 | 2.7 | 0.1 | 1.0 | 0.7 | 3.0 | 0.7 | 0.4 | 2.4 | 0.4 | 0.3 | 0.4 | 0.0 | 0.3 | 0.4 | 0.6 |
| NAPPA-Stigma visit 1 | 0.7 | 0.0 | 1.6 | 0.0 | 1.0 | 0.0 | 2.4 | 0.4 | 0.0 | 1.7 | 0.0 | 0.0 | 0.7 | 0.0 | 0.1 | - | - | 0.7 |
| NAPPA-Stigma visit X | 0.0 | - | 0.0 | 1.4 | - | - | 0.0 | 2.9 | - | - | - | 0.4 | - | - | - | - | - | 0.4 |
| NAPPA-Everyday Life baseline | 0.0 | 0.7 | 0.0 | 2.3 | 0.0 | 1.0 | 0.4 | 2.9 | 0.0 | 0.9 | 2.1 | 0.0 | 0.4 | 0.7 | 1.0 | 0.4 | 0.7 | 0.4 |
| NAPPA-Everyday Life visit 1 | 0.1 | 0.0 | 1.3 | 0.0 | 1.0 | 0.0 | 2.1 | 0.0 | 0.0 | 0.6 | 0.0 | 0.0 | 0.4 | 0.1 | 0.1 | - | - | 0.0 |
| NAPPA-Everyday Life visit X | 0.9 | - | 0.0 | 2.1 | - | - | 0.4 | 2.4 | - | - | - | 0.0 | - | - | - | - | - | 0.4 |
| DLQI Baseline | 7 | 7 | 10 | 16 | 13 | 4 | 5 | 17 | 13 | 8 | 23 | 22 | 7 | 21 | 25 | 28 | 18 | 6 |
| DLQI visit 1 | 0 | 0 | 0 | 7 | 2 | 1 | 0 | 1 | 0 | 3 | 0 | 0 | - | 4 | 1 | 13 | - | 0 |
| DLQI visit X | 13 | - | 2 | 8 | - | - | 1 | 16 | - | - | - | 11 | - | - | - | - | - | 1 |
| EQ-5D index value baseline | 0.79 | 0.86 | 0.79 | 0.75 | 0.86 | 0.86 | 0.86 | 0.76 | 0.77 | 0.69 | 0.75 | 0.79 | 0.86 | 0.70 | 0.82 | 0.63 | 0.73 | 1.00 |
| EQ-5D index value visit 1 | 1.00 | 1.00 | 1.00 | 0.86 | 0.74 | 1.00 | 1.00 | 0.69 | 1.00 | 1.00 | 0.73 | 0.86 | - | 1.00 | 0.86 | 0.64 | - | 1.00 |
| EQ-5D index value visit X | 0.70 | - | 0.79 | 0.86 | - | - | 0.80 | 0.80 | - | - | - | - | - | - | - | - | - | 1.00 |
| EQ-VAS baseline | - | 70 | 60 | 58 | 75 | 75 | 70 | 41 | 55 | 54 | 45 | 60 | 60 | 50 | 95 | 25 | 76 | 68 |
| EQ-VAS visit 1 | 75 | 70 | 100 | 75 | 75 | 92 | 97 | 75 | 100 | 45 | 86 | 90 | - | 79 | 40 | 20 | - | 97 |
| EQ-VAS visit X | 60 | - | 70 | 75 | - | - | 62 | 40 | - | - | - | 70 | - | - | - | - | - | 40 |
